# Book Review

**DOI:** 10.1120/jacmp.v2i2.2620

**Published:** 2001-03-01

**Authors:** Jim Smathers

**Affiliations:** ^1^ Department of Radiation Oncology B265 200 UCLA Medical Plaza Los Angeles CA 90095‐6951

## Abstract

**General Practice of Radiation Oncology Physics in the 21st Century**. Edited by Almon Shiu, Ph.D. and David Mellenberg Ph.D., 356 pp. Medical Physics Publishing, Madison, WI. 1998. ISBN: 0‐944838‐98‐7. Price: $75.00.

PACS number(s): 87.53.–j

The book is the hard copy published proceedings of year 2000 AAPM Summer School at Northern Illinois University. The papers are grouped into ten major sections and some 35 authors participated in preparing the material.

As with most AAPM Summer Schools, the tenor of the proceedings is to update the practicing medical physicist on the current status of the topical areas being addressed. Thus the title is a bit misleading in that there is minimal effort given to predicting the future. The scope of topics covered was broad and well done, with minimal use of equations. The topics covered are most efficiently described by listing the ten major sections.

- Dose Specification, Dose Prescription, and Dose Volume Histogram (DVH) Analysis
- Three Dimensional (3D) Treatment Planning (Photon Dose Algorithm and 3DRTP Process)
- Forward Treatment Planning
- Inverse Treatment Planning and Intensity‐Modulated Radiotherapy (IMRT)
- Calibration Protocol–Tg‐51
- Quality Assurance For Treatment Planning and Dose Delivery by 3DRTP, FRTP, and IMRT
- Dose Prescriptions and the TG‐43 Protocol
- New Implant Applications–Ultrasound Guided Prostate Implants and Endovascular Systems
- HDR Techniques
- Detectors for 2D or 3D Dosimetry Measurements

The quality of the paper used is typical for that used in proceedings and as such several figures are poorly reproduced. The text and diagrams are crisp and easily read.

For the clinical physicist initiating a program in one of the covered topical areas the book is a good starting place to scope the topic. Numerous references, some as recent as early 2000, are given to allow the reader to easily find more substantial papers on the details of the techniques.

For the experienced therapy physicist, a nice update on the current status of the field by authors that are actively developing the treatment techniques discussed.

